# Supplementary material for: Evaluation of outbreak response immunization in the control of pertussis using agent-based modeling
Source: PeerJ. 2016 Aug 18;4:e2337. doi: 10.7717/peerj.2337 (PMC4994102; doi:10.7717/peerj.2337)

**Supplementary Material for**

**Evaluation of outbreak response immunization in the control of pertussis using agent-based modeling**

**Table of content**

1. Supplementary table 1A. Distribution of individuals in the population by vaccine attitude: model input at the initialization
2. Supplementary table 1B. Probabilities of vaccination by attitude: model input at the initialization
3. Supplementary figure 1A. Auto-ORI algorithm overview.
4. Supplementary figure 1B. Auto-ORI algorithm – rating sub-outbreak levels
5. Supplementary figure 1C. Auto-ORI algorithm – evaluating/determining outbreak levels
6. Supplementary figure 2. 15-years cumulative incidence data for two reference populations: (A) Alberta Central Zone; (B) Alberta South Zone
7. Supplementary figure 3. Calculating waning immunity algorithm
8. Supplementary figure 4A. Illustration of vaccine-induced waning immunity output by years of simulation, 4-16 age group
9. Supplementary figure 4B. Illustration of natural infection-derived waning immunity output by years of simulation, 4-16 age group
10. Supplementary figure 5. Scatterplot illustrating differences in case counts in a single paired simulation run, all ages
11. Supplementary table 2A – Sensitivity analysis with vaccine coverage for dose 7 equals 27%. Number of pertussis cases averted and numbers needed to vaccinate by time periods after the outbreak-response immunization campaign and by age groups: modeling-generated results
12. Supplementary figure 6A – Sensitivity analysis with vaccine coverage for dose 7 equals 27%. Boxplot of pertussis cases averted over time, up to 10 years after outbreak response immunization campaign: All ages (A), infants under 1 year of age (B) and adolescents 10-14 years of age (C)
13. Supplementary table 2B – Sensitivity analysis with τ value increased to 3 for individuals born before 1997. Number of pertussis cases averted and numbers needed to vaccinate by time periods after the outbreak-response immunization campaign and by age groups: modeling-generated results
14. Supplementary figure 6B – Sensitivity analysis with τ value increased to 3 for individuals born before 1997. Boxplot of pertussis cases averted over time, up to 10 years after outbreak response immunization campaign: All ages (A), infants under 1 year of age (B) and adolescents 10-14 years of age (C)
15. Supplementary table 2C – Sensitivity analysis with α value increased to 10. Number of pertussis cases averted and numbers needed to vaccinate by time periods after the outbreak-response immunization campaign and by age groups: modeling-generated results
16. Supplementary figure 6C – Sensitivity analysis with α value increased to 10. Boxplot of pertussis cases averted over time, up to 10 years after outbreak response immunization campaign: All ages (A), infants under 1 year of age (B) and adolescents 10-14 years of age (C)
17. Supplementary table 2D – Sensitivity analysis: Restricting ORI eligibility to those who did not receive vaccine within last 6 months. Number of pertussis cases averted and numbers needed to vaccinate by time periods after the outbreak-response immunization campaign and by age groups: modeling-generated results
18. Supplementary figure 6D Sensitivity analysis: Restricting ORI eligibility to those who did not receive vaccine within last 6 months. Boxplot of pertussis cases averted over time, up to 10 years after outbreak response immunization campaign: All ages (A), infants under 1 year of age (B) and adolescents 10-14 years of age (C)
19. Supplementary table 2E. Sensitivity analysis: Enhancing boosting effect. Number of pertussis cases averted and numbers needed to vaccinate by time periods after the outbreak-response immunization campaign and by age groups: modeling-generated results
20. Supplementary figure 6E. Sensitivity analysis: Enhancing boosting effect. Boxplot of pertussis cases averted over time, up to 10 years after outbreak response immunization campaign: All ages (A), infants under 1 year of age (B) and adolescents 10-14 years of age (C)
21. Supplementary table 2F. Multi-way sensitivity analysis: τ value increased to 3 for individuals born before 1997 and α value increased to 10. Number of pertussis cases averted and numbers needed to vaccinate by time periods after the outbreak-response immunization campaign and by age groups: modeling-generated results
22. Supplementary figure 6F. Multi-way sensitivity analysis: τ value increased to 3 for individuals born before 1997 and α value increased to 10. Boxplot of pertussis cases averted over time, up to 10 years after outbreak response immunization campaign: All ages (A), infants under 1 year of age (B) and adolescents 10-14 years of age (C)

**Supplementary table 1A. Distribution of individuals in the population by vaccine attitude: model input at the initialization**

|  | Vaccination attitude | | |
| --- | --- | --- | --- |
|  | Acceptor | Hesitant | Rejecter |
| Percentage in the population | 50% | 40% | 10% |

**Supplementary table 1B. Probabilities of vaccination by attitude: model input at the initialization**

| Dose | Acceptor | Hesitant | Rejecter |
| --- | --- | --- | --- |
| 1 | 0.95 | 0.6 | 0 |
| 2 | 0.95 | 0.6 | 0 |
| 3 | 0.95 | 0.6 | 0 |
| 4 | 0.9 | 0.6 | 0 |
| 5 | 0.9 | 0.6 | 0 |
| 6 | 0.9 | 0.6 | 0 |
| 7 | 0.1 | 0.05 | 0 |

Table 1A demonstrates the distribution of vaccination attitudes in the simulated population. Vaccination attitudes for children are assumed to be those of their parents. Table 1B shows final values of the dose-specific vaccination probabilities based on vaccine attitudes. Values were varied during the calibration process to derive model-generated dose-specific vaccine coverage judged to be comparable with reference vaccine coverage values. References values were derived from Alberta Health, Public Health Ontario and Public Health Agency of Canada.

**Supplementary figure 1A. Auto-ORI algorithm overview.**


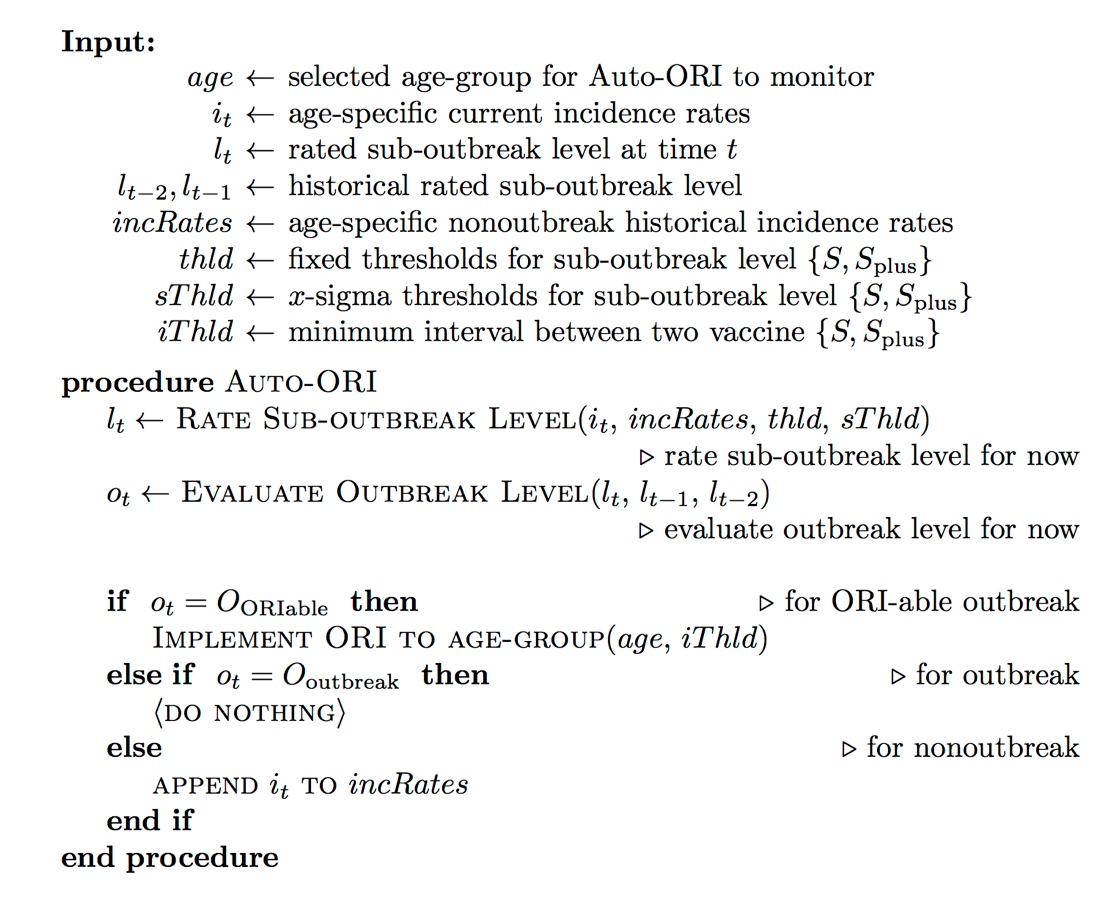


We considered ORI to be supplementary immunization delivered to a specified age-group based on the magnitude of the outbreak. Therefore the decision to implement ORI was based in “real time” on analysis of current and historical incidence rates for each age-group separately. The auto-ORI algorithm in our model is based on evaluation/determination of the outbreak level, while the outbreak level is further decided based on historical and current sub-outbreak levels as demonstrated in supplementary figure 1A above.

**Supplementary figure 1B. Auto-ORI algorithm – rating sub-outbreak levels.**


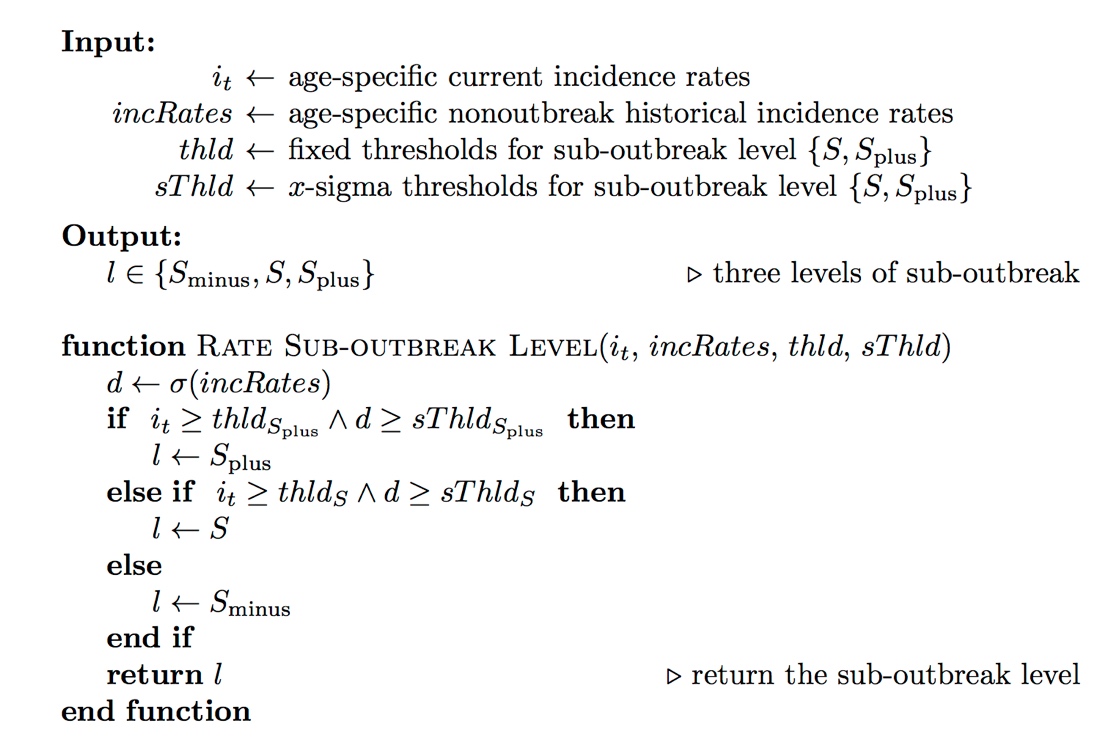


We rated sub-outbreak levels/states by examining past five years’ monthly incidence rates for the specific age-groups and used both fixed (but modifiable) thresholds and standard deviations above 5-year mean as criteria to classify monthly incidence rates into S and S+ states, as shown in supplementary figures 1B.

**Supplementary figure 1C. Auto-ORI algorithm – evaluating/determining outbreak levels**


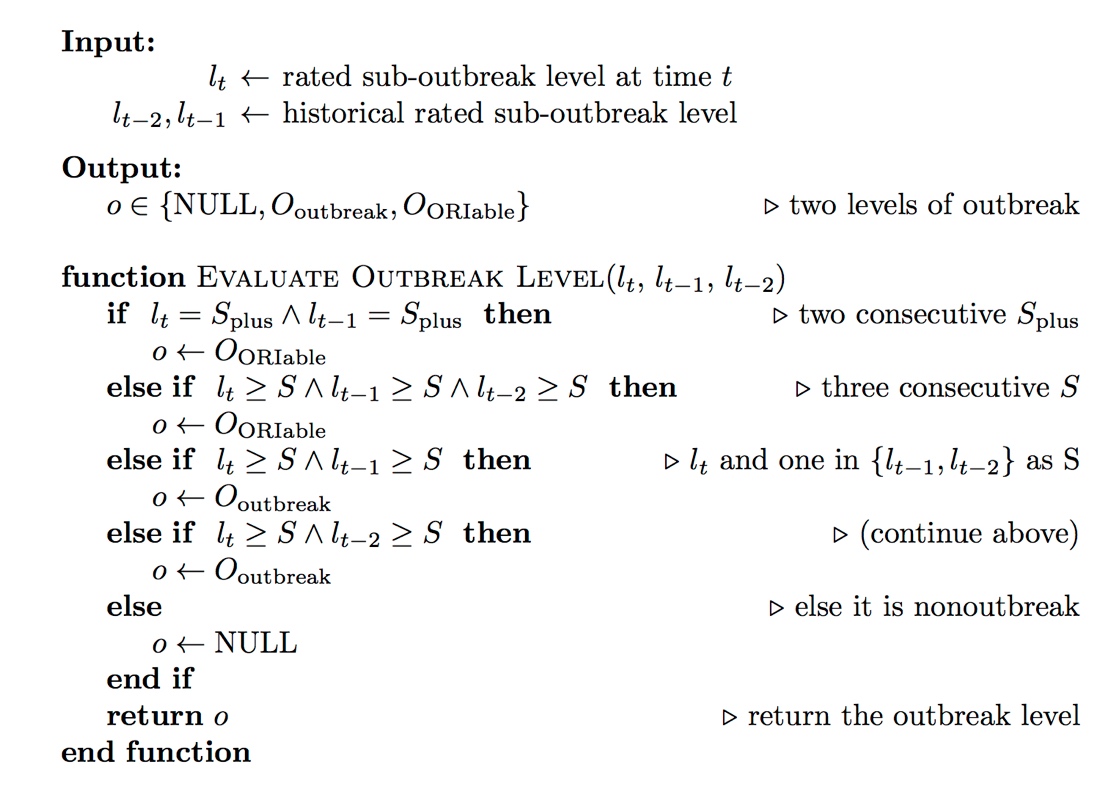


We classified potential outbreaks into three levels: non-outbreak, outbreak and outbreak triggering ORI (ORI-able outbreak), based on the magnitude and duration of monthly incidence rates, which were expressed as sequences (monthly runs) of sub-outbreak levels as shown in supplementary figure 1C. The outbreak was defined as at least two consecutive S states, while ORI-able outbreak was defined as three consecutive S states or two consecutive S+ states. S+ state would equal to the S state for the purpose of defining outbreaks but not vice versa (e.g., S, S+ sequence would be classified as outbreak but not ORI-able outbreak; adding either S or S+ state in the following month would upgrade this outbreak to the ORI-able outbreak).

**Supplementary figure 2. 15-years cumulative incidence^¶^ data for two reference populations: (A) Alberta Central Zone; (B) Alberta South Zone**


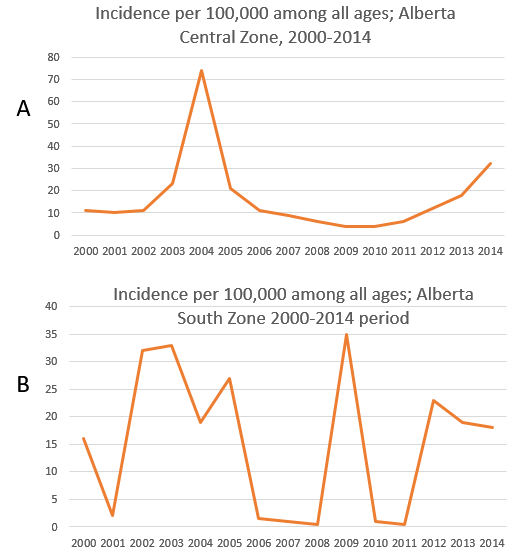


^¶^ 15-years cumulative incidence was up-scaled to 30-years cumulative incidence for calibration purposes. For Central zone – up-scaled 30-years cumulative incidence is 504 per 100,000; for South zone – up-scaled 30-years cumulative incidence is 456 per 100,000. Data derived from Alberta Heath, Interactive Health Data Application, 2015.

Supplementary figure 2 demonstrates 15-years cumulative incidence of pertussis in two jurisdictions in Alberta, Canada: Central and South zones. These jurisdictions were selected as reference based on having comparable population to the population size simulated in the model. As only 15 years of reliable data were available, 15-years cumulative incidence was up-scaled to 30 years. Two reference population presented here have comparable cumulative incidence, but dis-similar shape of epidemiological curves (e.g. Central zone had one year with a larger peak while South zone had several smaller peaks). The variability of epidemiologic curve shapes (distribution of incidence over time) is represented in our model simulations. The implication of having such variability is that the simulation resulting in a higher peak is more likely to trigger outbreak response (as may happen in reality), while smaller-scale outbreaks will be managed without outbreak response immunization.

**Supplementary figure 3. Calculating vaccine-induced waning immunity algorithm.**


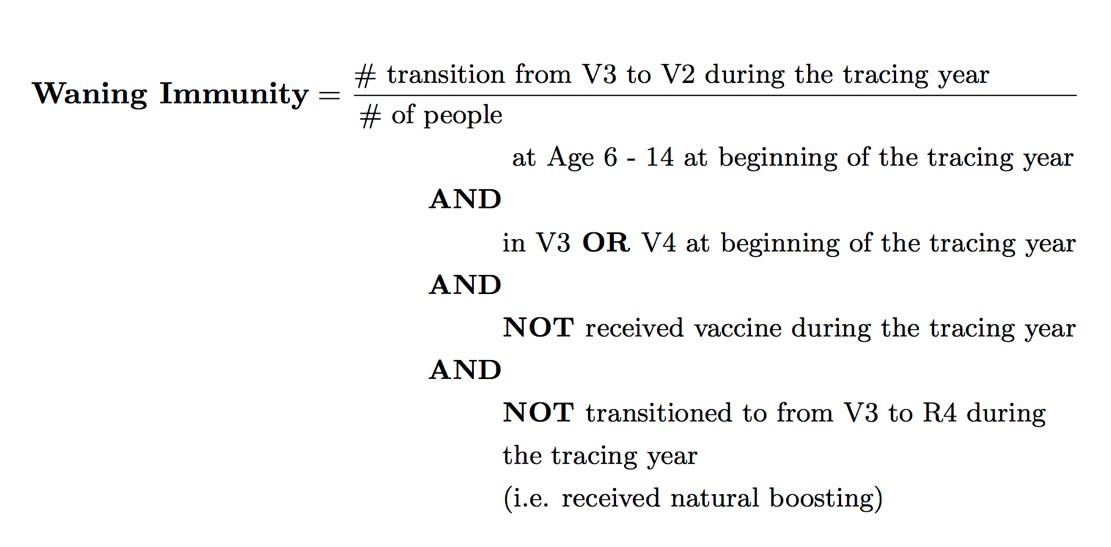


Vaccine-induced waning immunity was calculated by identifying qualifying group of individuals (age group) at the beginning of tracing year and then count individuals who transitioned from V3 state to V2 state during a tracing year while excluding those who received natural boosting (by virtue of contracting disease). Waning immunity was expressed as a percentage (or fraction of population) per year.

**Supplementary figure 4A. Illustration of vaccine-induced waning immunity^§^ output by years of simulation, 4-16 age group**


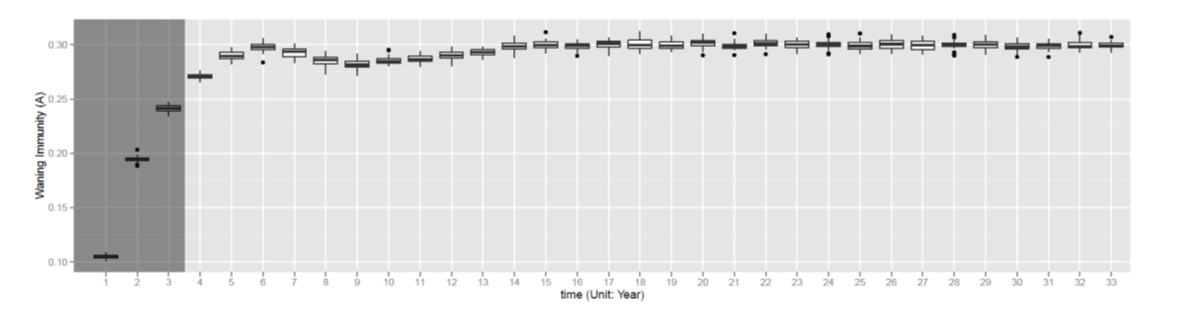
**Supplementary figure 4B. Illustration of natural infection-derived waning immunity^§^ output by years of simulation, 4-16 age group**
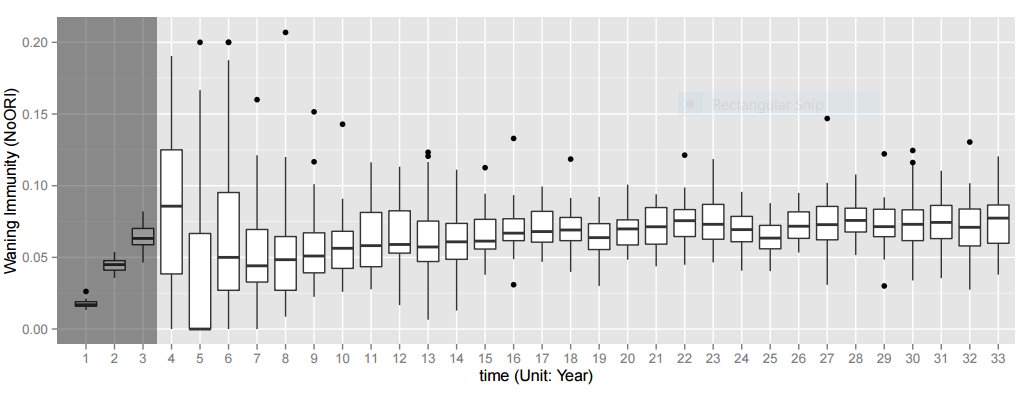
**^§^** Based on “No-ORI” runs [no outbreak response immunization] and expressed as annual percentage transition from the V3 or R3 state to V2 or R2 state

Supplementary figures 4A and 4B demonstrate distribution of vaccine-induced and natural infection-derived waning immunity over time. For each given year of 30-year study period, distribution reflects different values of waning immunity in different simulations. There is a smaller fluctuation of vaccine-induced waning immunity in comparison to natural disease-derived waning immunity over time. This is likely be explained by more predictable vaccination patterns for agents in our model, while natural infection immunity is less predictable due to outbreaks’ occurrence.

**Supplementary figure 5. Scatterplot illustrating differences^§^ in case counts in a single paired simulation run, all ages^¶^**


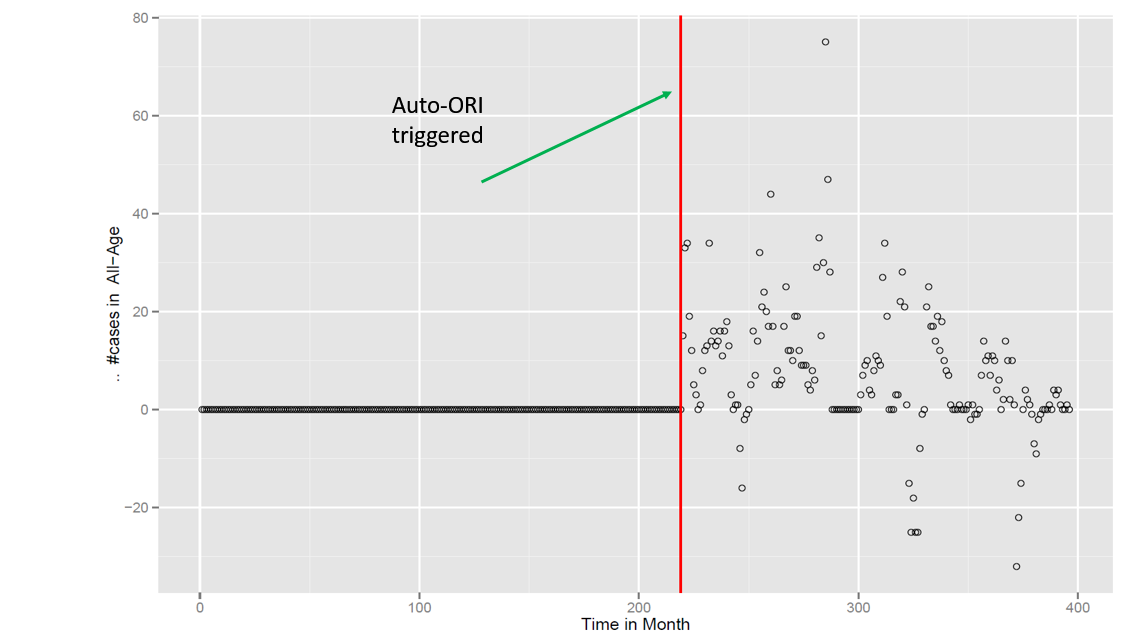


**^§^** Difference is between count of cases in “No-ORI” runs [no outbreak response immunization] versus ORI [outbreak response immunization triggered] run. Positive number [dots above zero line on *y*-axis] represent “cases averted”, while negative number [dots below zero line on *y*-axis] represent “cases added” as a result of ORI

^¶^ Case count for all ages

Supplementary figure 5 illustrates a representative example of the single individual paired “No-ORI” – “ORI” simulation. The “No-ORI” simulation serves as a control group. The timing of triggered-ORI is shown as a vertical red line. The figure captures the difference in the number of pertussis cases occurring after the implementation of the ORI. Prior to ORI implementation the number of cases in both “No-ORI” and “ORI” simulations are identical and both these simulations are run from the same randomly-selected seed. The *y-axis* represents the difference in the number of cases between paired “No-ORI” and “ORI” runs with positive number (i.e. dots above horizontal zero line) representing cases averted (greater number of cases during “No-ORI” in comparison to “ORI” run), while negative number (i.e. dots below horizontal zero line) indicates additional cases occurring as a results of having ORI (“harmful” effect of ORI).

Supplementary tables 2A through 2F and figures 6A to 6F demonstrate results of sensitivity analyses performed. Numbers of cases averted reported in these tables are compared to those reported in the main simulation to check for the robustness of the model and its sensitivity to changes in various parameters.

**Supplementary table 2A. Sensitivity analysis with vaccine coverage for dose 7 equals 27%. Number of pertussis cases averted and numbers needed to vaccinate by time periods after the outbreak-response immunization campaign and by age groups: modeling-generated results**

| Age groups | Post-outbreak-response immunization period, years | Number of cases averted* | Minimum number needed to vaccinate^§^ | Maximum number needed to vaccinate^§^ |
| --- | --- | --- | --- | --- |
| All ages | 1 | 119 | 176 | 228 |
| All ages | 3 | 256 | 82 | 106 |
| All ages | 10 | 410 | 51 | 66 |
| Under 1 year | 1 | 5 | 4067 | 5254 |
| Under 1 year | 3 | 12 | 1789 | 2311 |
| Under 1 year | 10 | 20 | 1038 | 1341 |
| 10-14 years old | 1 | 51 | 409 | 529 |
| 10-14 years old | 3 | 102 | 205 | 265 |
| 10-14 years old | 10 | 156 | 134 | 173 |

*p<0.00001 for all groups of comparisons of counts of cases in outbreak-response immunization (ORI) versus no-ORI simulations, one-way Mann Whitney U test;

^§^ Number needed to vaccinate (NNV) was calculated directly from the model by dividing a number of vaccinations delivered during the ORI by a number of cases averted in a respective group. NNV only applies to a current model and for a given population size.

**Supplementary figure 6A. Sensitivity analysis with vaccine coverage for dose 7 equals 27%. Boxplot of pertussis cases averted over time, up to 10 years after outbreak response immunization campaign: All ages (A), infants under 1 year of age (B) and adolescents 10-14 years of age (C)**


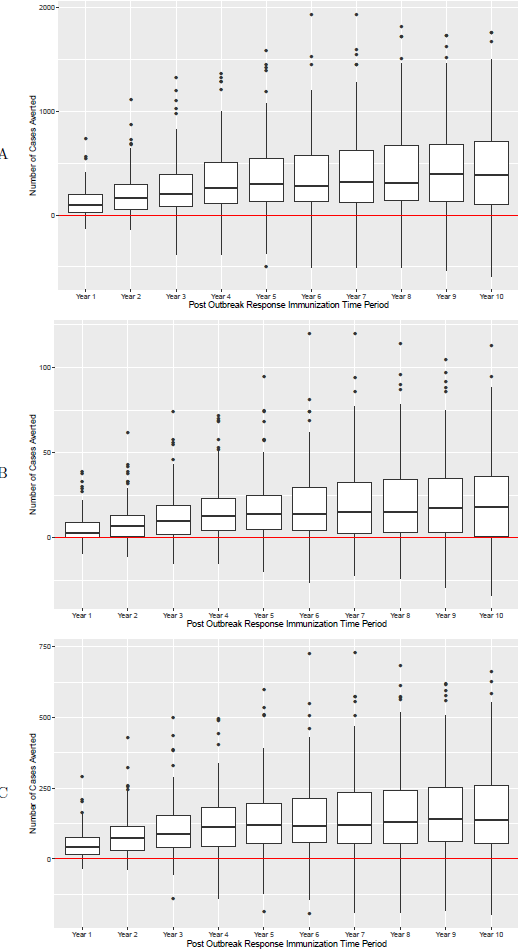


**Supplementary table 2B. Sensitivity analysis with τ value^¶^ increased to 3 for individuals born before 1997. Number of pertussis cases averted and numbers needed to vaccinate by time periods after the outbreak-response immunization campaign and by age groups: modeling-generated results**

| Age groups | Post-outbreak-response immunization period, years | Number of cases averted* | Minimum number needed to vaccinate^§^ | Maximum number needed to vaccinate^§^ |
| --- | --- | --- | --- | --- |
| All ages | 1 | 112 | 188 | 245 |
| All ages | 3 | 228 | 92 | 120 |
| All ages | 10 | 409 | 51 | 67 |
| Under 1 year | 1 | 5 | 4181 | 5448 |
| Under 1 year | 3 | 10 | 2005 | 2612 |
| Under 1 year | 10 | 20 | 1041 | 1356 |
| 10-14 years old | 1 | 48 | 434 | 565 |
| 10-14 years old | 3 | 92 | 229 | 298 |
| 10-14 years old | 10 | 156 | 135 | 175 |

**^¶^** τ value represents the transition between V states in the model. Increasing its value reduces vaccine-induced waning immunity and increase protection. This was applied only to individuals born before 1997, who were recipients of whole-cell pertussis vaccine.

*p<0.00001 for all groups of comparisons of counts of cases in outbreak-response immunization (ORI) versus no-ORI simulations, one-way Mann Whitney U test;

^§^ Number needed to vaccinate (NNV) was calculated directly from the model by dividing a number of vaccinations delivered during the ORI by a number of cases averted in a respective group. NNV only applies to a current model and for a given population size.

**Supplementary figure 6B. Sensitivity analysis with τ value increased to 3 for individuals born before 1997. Boxplot of pertussis cases averted over time, up to 10 years after outbreak response immunization campaign: All ages (A), infants under 1 year of age (B) and adolescents 10-14 years of age (C)**


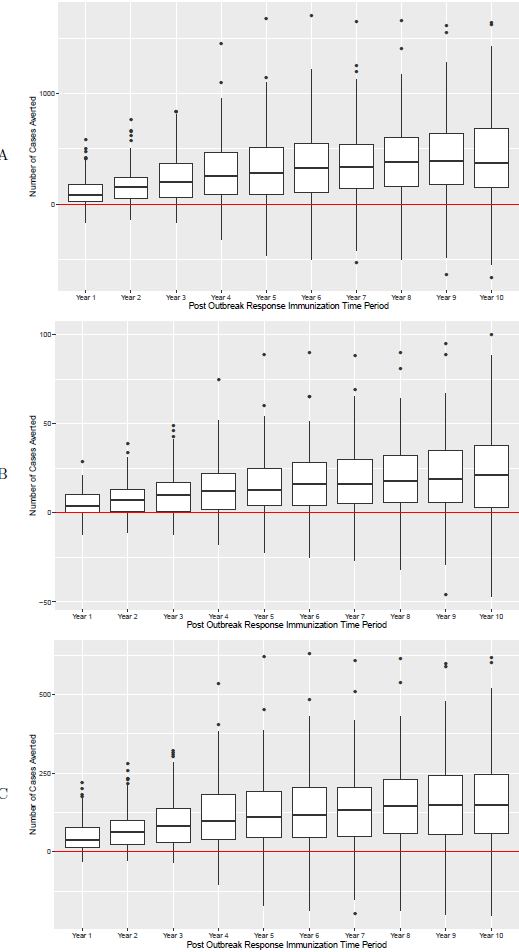


**Supplementary table 2C. Sensitivity analysis with α value^¶^ increased to 10. Number of pertussis cases averted and numbers needed to vaccinate by time periods after the outbreak-response immunization campaign and by age groups: modeling-generated results**

| Age groups | Post-outbreak-response immunization period, years | Number of cases averted* | Minimum number needed to vaccinate^§^ | Maximum number needed to vaccinate^§^ |
| --- | --- | --- | --- | --- |
| All ages | 1 | 148 | 143 | 183 |
| All ages | 3 | 262 | 81 | 103 |
| All ages | 10 | 422 | 50 | 64 |
| Under 1 year | 1 | 7 | 2998 | 3850 |
| Under 1 year | 3 | 13 | 1661 | 2132 |
| Under 1 year | 10 | 21 | 1016 | 1305 |
| 10-14 years old | 1 | 61 | 348 | 447 |
| 10-14 years old | 3 | 104 | 203 | 261 |
| 10-14 years old | 10 | 161 | 131 | 168 |

**^¶^** α value represents the transition between R states in the model. Increasing its value reduces natural infection-derived waning immunity and increase protection.

*p<0.00001 for all groups of comparisons of counts of cases in outbreak-response immunization (ORI) versus no-ORI simulations, one-way Mann Whitney U test;

^§^ Number needed to vaccinate (NNV) was calculated directly from the model by dividing a number of vaccinations delivered during the ORI by a number of cases averted in a respective group. NNV only applies to a current model and for a given population size.

**Supplementary figure 6C. Sensitivity analysis with α value increased to 10. Boxplot of pertussis cases averted over time, up to 10 years after outbreak response immunization campaign: All ages (A), infants under 1 year of age (B) and adolescents 10-14 years of age (C)**


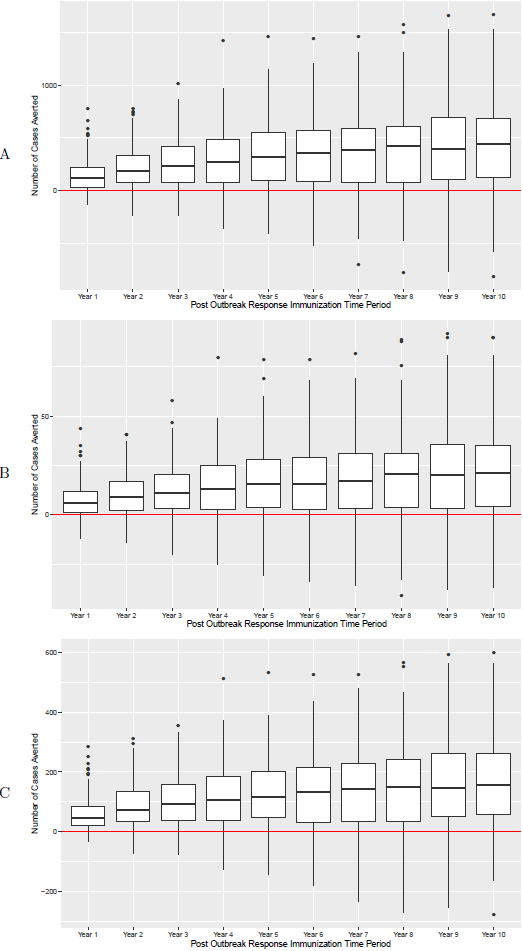


**Supplementary table 2D. Sensitivity analysis: Restricting ORI eligibility to those who did not receive vaccine within last 6 months. Number of pertussis cases averted and numbers needed to vaccinate by time periods after the outbreak-response immunization campaign and by age groups: modeling-generated results**

| Age groups | Post-outbreak-response immunization period, years | Number of cases averted* | Minimum number needed to vaccinate^§^ | Maximum number needed to vaccinate^§^ |
| --- | --- | --- | --- | --- |
| All ages | 1 | 108 | 184 | 235 |
| All ages | 3 | 241 | 82 | 106 |
| All ages | 10 | 429 | 46 | 60 |
| Under 1 year | 1 | 4 | 4555 | 5836 |
| Under 1 year | 3 | 10 | 1910 | 2448 |
| Under 1 year | 10 | 21 | 949 | 1216 |
| 10-14 years old | 1 | 47 | 422 | 541 |
| 10-14 years old | 3 | 96 | 208 | 266 |
| 10-14 years old | 10 | 162 | 123 | 158 |

*p<0.00001 for all groups of comparisons of counts of cases in outbreak-response immunization (ORI) versus no-ORI simulations, one-way Mann Whitney U test;

^§^ Number needed to vaccinate (NNV) was calculated directly from the model by dividing a number of vaccinations delivered during the ORI by a number of cases averted in a respective group. NNV only applies to a current model and for a given population size.

**Supplementary figure 6D. Sensitivity analysis: Restricting ORI eligibility to those who did not receive vaccine within last 6 months. Boxplot of pertussis cases averted over time, up to 10 years after outbreak response immunization campaign: All ages (A), infants under 1 year of age (B) and adolescents 10-14 years of age (C)**


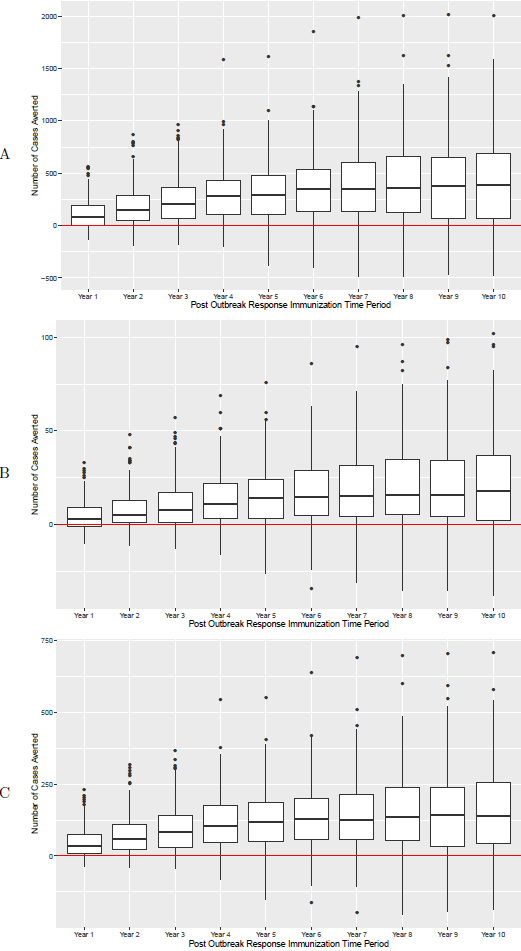


**Supplementary table 2E. Sensitivity analysis: Enhancing boosting effect^¶^. Number of pertussis cases averted and numbers needed to vaccinate by time periods after the outbreak-response immunization campaign and by age groups: modeling-generated results**

| Age groups | Post-outbreak-response immunization period, years | Number of cases averted* | Minimum number needed to vaccinate^§^ | Maximum number needed to vaccinate^§^ |
| --- | --- | --- | --- | --- |
| All ages | 1 | 41 | 521 | 667 |
| All ages | 3 | 93 | 227 | 290 |
| All ages | 10 | 148 | 143 | 183 |
| Under 1 year | 1 | 1** | 17238 | 22050 |
| Under 1 year | 3 | 4 | 5572 | 7127 |
| Under 1 year | 10 | 6 | 3536 | 4523 |
| 10-14 years old | 1 | 19 | 1110 | 1420 |
| 10-14 years old | 3 | 40 | 537 | 686 |
| 10-14 years old | 10 | 60 | 351 | 449 |

**^¶^** In this scenario, booster immunization for an individual in lower V states would lead to a V4 state (full protection) without transitioning through V2 and V3 states as is in the main experiment

*p<0.00001 for all groups of comparisons of counts of cases in outbreak-response immunization (ORI) versus no-ORI simulations, one-way Mann Whitney U test;

** p=0.12

^§^ Number needed to vaccinate (NNV) was calculated directly from the model by dividing a number of vaccinations delivered during the ORI by a number of cases averted in a respective group. NNV only applies to a current model and for a given population size.

**Supplementary figure 6E. Sensitivity analysis: Enhancing boosting effect. Boxplot of pertussis cases averted over time, up to 10 years after outbreak response immunization campaign: All ages (A), infants under 1 year of age (B) and adolescents 10-14 years of age (C)**


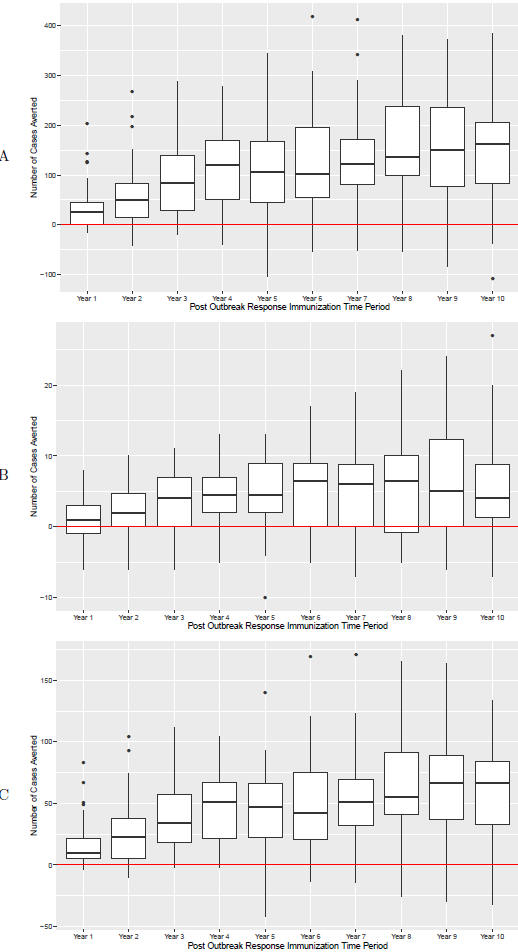


**Supplementary table 2F. Multi-way sensitivity analysis: τ value increased to 3 for individuals born before 1997^†^ and α value increased to 10^¶^. Number of pertussis cases averted and numbers needed to vaccinate by time periods after the outbreak-response immunization campaign and by age groups: modeling-generated results**

| Age groups | Post-outbreak-response immunization period, years | Number of cases averted* | Minimum number needed to vaccinate^§^ | Maximum number needed to vaccinate^§^ |
| --- | --- | --- | --- | --- |
| All ages | 1 | 130 | 162 | 209 |
| All ages | 3 | 229 | 92 | 119 |
| All ages | 10 | 378 | 56 | 72 |
| Under 1 year | 1 | 6 | 3590 | 4623 |
| Under 1 year | 3 | 10 | 2012 | 2591 |
| Under 1 year | 10 | 18 | 1164 | 1498 |
| 10-14 years old | 1 | 54 | 390 | 502 |
| 10-14 years old | 3 | 90 | 235 | 302 |
| 10-14 years old | 10 | 143 | 148 | 191 |

**^†^** τ value represents the transition between V states in the model. Increasing its value reduces vaccine-induced waning immunity and increase protection. This was applied only to individuals born before 1997, who were recipients of whole-cell pertussis vaccine.

**^¶^** α value represents the transition between R states in the model. Increasing its value reduces natural infection-derived waning immunity and increase protection.

*p<0.00001 for all groups of comparisons of counts of cases in outbreak-response immunization (ORI) versus no-ORI simulations, one-way Mann Whitney U test;

^§^ Number needed to vaccinate (NNV) was calculated directly from the model by dividing a number of vaccinations delivered during the ORI by a number of cases averted in a respective group. NNV only applies to a current model and for a given population size.

**Supplementary figure 6F. Multi-way sensitivity analysis: τ value increased to 3 for individuals born before 1997 and α value increased to 10. Boxplot of pertussis cases averted over time, up to 10 years after outbreak response immunization campaign: All ages (A), infants under 1 year of age (B) and adolescents 10-14 years of age (C)**


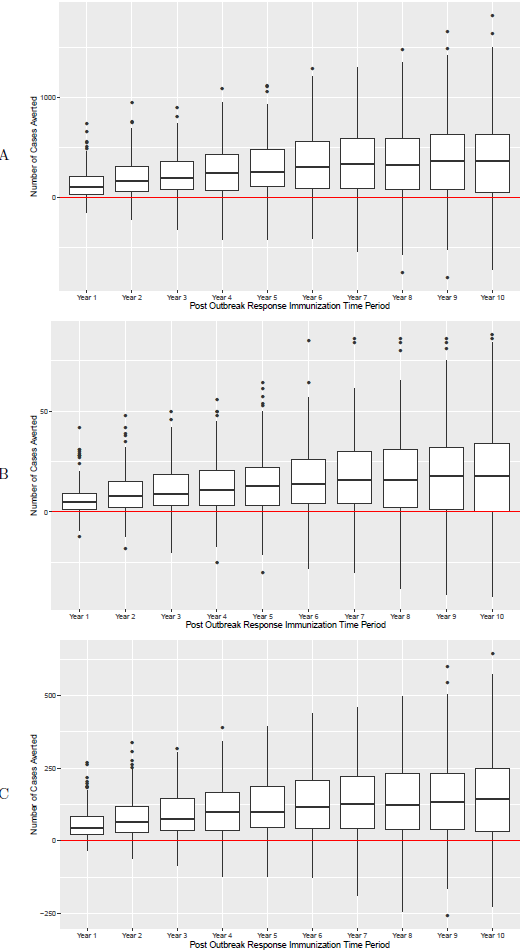

Supplement: Supplemental Information 1 [file peerj-04-2337-s001.docx]
